# Retrospective Analysis of the Impact of SARS-CoV-2 (COVID-19) on Pregnancy and Neonatal Outcomes

**DOI:** 10.1155/2024/1177119

**Published:** 2024-08-06

**Authors:** Leyla Sero, Nilufer Okur, Duygu Tunçel, Mehmet Nur Talay, Mustafa Fırat Aydın, Suleyman Cemil Oglak

**Affiliations:** ^1^ Neonatology Department Diyarbakır Gazi Yaşargil Training and Research Hospital, Diyarbakır, Turkey; ^2^ Obstetrics and Gynaecology Department Diyarbakır Gazi Yaşargil Training and Research Hospital, Diyarbakır, Turkey

**Keywords:** COVID-19, mortality, neonate, pregnancy

## Abstract

**Background:** The novel coronavirus disease 2019 (COVID-19) was more devastating in people with comorbidities such as advanced age and immunodeficiency. Another group affected by COVID-19 was pregnant women. Immunological changes during pregnancy and conditions such as gestational diabetes and pre-eclampsia that occur during pregnancy also have effects on the fetus. The aim of this study was to analyze the effects of PCR-proven COVID-19 infection during pregnancy on fetus and newborn.

**Methods:** Between December 2019 and October 2021, data from pregnant women with COVID-19 symptoms or a history of contact with people with COVID-19, infected with PCR-proven COVID-19 virus, were analyzed retrospectively. Clinical and laboratory data of pregnant women were analyzed. Death data associated with COVID-19 were evaluated. Clinical and laboratory findings of newborns related to COVID-19 and mortality data related to COVID-19 were recorded. The study received approval from the Gazi Yasargil Training and Research Hospital ethics committee (09.07.2021/853).

**Results:** We evaluated 327 pregnant women who were followed up in our hospital and whose deliveries ended in live birth, stillbirth, miscarriage, or curettage. One hundred eighty-five (56.6%) of the pregnant women had at least one COVID-19-related symptom. We evaluated the data of 306 live births, 21 intrauterine fetal deaths, and 13 postnatal deaths. Among the postnatal deaths, five infants succumbed directly due to COVID-19 infection. A total of 23 live-born babies (7.5%) were classified as small for gestational age (SGA), while 80 babies (26.1%) were born before 37 weeks of gestation, and 32 babies (10.4%) were born before 32 weeks. Cord blood gas analysis revealed that 19 infants (6.3%) had pH < 7 and base excess (BE) < −12. The rate of perinatal asphyxia was significantly higher in babies born to mothers who did not survive (*p* = 0.027). A considerable number of infants, 119 (40.3%), were admitted to the neonatal intensive care unit (NICU). Among the seven infants with positive PCR results admitted to the NICU, five (4.2%) did not survive.

**Conclusion:** While COVID-19 infection in pregnancy seriously affects mortality and morbidity in pregnant women, it also causes mortality and morbidity on the fetus.

## 1. Introduction

Pregnancy is considered a risk factor for serious illness from coronavirus disease 2019 (COVID-19) [1, 2]. Pregnancy is a special condition that can have significant effects on a woman's body's biological systems. Although physiological, mechanical, and immunological changes in pregnancy can potentially affect susceptibility to COVID-19 during pregnancy, limited data are available. Many studies have reported the prevalence of SARS-CoV-2 infection among pregnant people presenting in labor and delivery, with estimates varying between 3% and 20% [3–6].

Although SARS-CoV-2 rarely appears to be transmitted to the fetus via the placenta, there is evidence that SARS-CoV-2 infection during pregnancy is associated with a number of adverse pregnancy outcomes. A meta-analysis found an increased risk of pre-eclampsia, preterm birth, and stillbirth in pregnant people with SARS-CoV-2 infection compared to those without SARS-CoV-2 infection [7].

Although a few cases of intrauterine SARS-CoV-2 transmission have been documented, transmission appears to be rare. Most SARS-CoV-2 infections identified among infants after birth are due to exposure to infected caregivers. However, it has been observed that the risk of late postnatal transmission increases when babies are not separated from their infected mothers after birth [8].

In this study, we aimed to investigate the effects of PCR-confirmed COVID-19 infection on the fetus and newborn baby during pregnancy.

## 2. Materials and Methods

Between December 2019 and October 2021, the data of women infected with the PCR-proven COVID-19 virus, from pregnant women followed in the reference gynecology and obstetrics clinic, were retrospectively analyzed. Data on patients were obtained from the hospital database.

Demographic data of pregnant women, comorbidities (diabetes, hypertension, and eclampsia), COVID-19-related symptoms, findings related to intensive care hospitalization (mechanical ventilator, noninvasive ventilation, oxygen requirement, and thorax computed tomography findings), and maternal death data associated with COVID-19 were evaluated.

Once more, data regarding birth outcomes, miscarriage or stillbirth occurrences, mode of delivery, necessity for baby resuscitation, preterm birth, and intrauterine growth retardation were examined.

Although COVID-19 PCR was positive during pregnancy, cases whose control was continued in other hospitals and whose pregnant-baby data were missing were not included in the study.

Newborn babies, prematurity status, first and fifth minute Apgar scores, cord blood gas analyses of risky babies, birth weight percentiles, hospitalization in the neonatal intensive care unit (NICU), COVID-19 PCR results, clinical and laboratory findings related to COVID-19, and COVID-19-associated mortality data were recorded.

The need for resuscitation, general appearance, pulse rate, grimace, activity, respiratory pattern, and APGAR scores have been recorded 1 and 5 min after the birth of the babies. Perinatal asphyxia was defined as pH < 7 and base excess (BE) < −12 in cord blood gas.

### 2.1. Management of COVID-19 in the Delivery Room and NICU

The deliveries occurred in a negative pressure isolation room and the neonates were separated from their mothers immediately after birth for further observation and treatment. Symptomatic and asymptomatic neonates born to mothers with suspected or confirmed COVID-19, regardless of the mother's symptoms, have been tested at approximately 24 h of age. If initial test results were negative, or not available, the tests were repeated at 48 h of age.

Permission was obtained from the Local Ethics Committee (09.07.2021/853).

Written consent was not obtained because our study was retrospective.

Statistical analyses were conducted using the SPSS statistical software for Windows, V.22.0 (SPSS, Chicago, Illinois, United States). The Kolmogorov–Smirnov test was used to test for the normality of data. Chi-square test will be applied for the analysis of the data. The median (minimum–maximum) is used for nonnormally distributed variables. A *p* value of less than 0.05 was considered significant for the statistical tests.

## 3. Results

Between December 2019 and October 2021, the data of 394 pregnant women with PCR-proven COVID-19 infection, we evaluated 327 pregnant women who were followed up in our hospital and whose deliveries ended in live birth, stillbirth, miscarriage, or curettage.

### 3.1. Demographic, Clinical, and Laboratory Characteristics of Pregnant With COVID-19

One hundred eighty-five (56.6%) of the pregnant women had at least one COVID-19-related symptom. The most common symptoms were fever (58.2%), cough (48%), and myalgia (32%). Other common symptoms included headache, fatigue, diarrhea, difficulty breathing, and chest pain. Thoracic computed tomography of ten cases included COVID-19 findings. Fifty-three (16.2%) pregnant women were over 35 years old. Intrauterine infant loss was higher in pregnant women over the age of 35 (8 (15%) years and 13 (4.7%), *p* = 0.011, respectively) years.

Fifty-four (16.5%) of the pregnant women were hospitalized in the intensive care unit, and 19 pregnant women needed mechanical ventilators. In the intensive care unit, ritonavir–lopinavir, favipiravir, dexamethasone, and enoxaparin treatments were given in various combinations. one hundred fourteen cases had comorbidities. There was pre-eclampsia in 25 (7.6%) cases and gestational diabetes in 10 (3%) cases. The presence of symptoms was not different in patients with diabetes than those without (*p* = 0.34). The rates of intensive care requirement and the presence of at least one symptom in pre-eclamptic pregnant women were similar to other pregnant women (*p* = 0.12 and *p* = 0.39, respectively). The number of 1st trimester abortions was eight (2.4%), 2nd trimester infant deaths were 10 (0.3%), and 3rd trimester infant deaths were 12 (3.7%).

Nonstress test (NST) abnormal trace was observed in 23.2% of the pregnant women. Cesarean section and prematurity rates were 43.7% and 26.7%, respectively. Fifty-four of 327 mothers (16.5%) were admitted to an intensive care unit, among whom 11 died (3.4%). Clinical and laboratory characteristics of pregnant women with COVID-19 PCR (+) are summarized in Table 1.

Twenty-two (6.7%) pregnancies resulted in stillbirth or abortion. A total of 11 (3.7%) pregnant deaths occurred. The median age of the mothers who died was 33 (IQR 25–75; 27–35) years. No statistical difference was found when the intrauterine infant deaths of pregnant women whose thorax tomography was compatible with COVID-19 and those who were normal were compared (5% and 6%, respectively, *p* = 1).

Group 1 includes women who died during the COVID-19 study period.

Group 2 is defined as pregnant women who survived COVID-19.

A comparison of the clinical and demographic characteristics of the groups is shown in Table 2. Of the mothers with COVID-19 PCR (+), 209 were detected in the third trimester, 21 in the second trimester, and 67 in the first trimester.

### 3.2. Demographic, Clinical, and Laboratory Characteristics of Neonates

Data from a total of 306 live births, 21 intrauterine dead fetuses, and 13 postnatal deaths were evaluated. Five of the babies who died postnatally died directly due to COVID-19 infection. Twenty-three (7.5%) of live-born babies were small for gestational age (SGA). Eighty (26.1%) babies were born under 37 weeks, and 32 (10.4%) babies were born under 32 weeks.

The cord blood gas sample was pH < 7 and BE < −12 (3.4%) in 19 (6.3%) infants. The perinatal asphyxia rate was significantly higher in babies of lost pregnant women (*p* = 0.027).

The birth weight of babies born to deceased mothers was lower than the birth weight of babies born to living mothers (*p* = 0.01) (Table 2). When a correlation analysis was performed between maternal age and infant asphyxia, no correlation was found between maternal age and pH and BE in cord blood gas (*r* = −0.062 and *p* = 0.52 and *r* = −0.052 and *p* = 0.64, respectively).

A total of 119 (40.3%) infants were admitted to the NICU. In the first months of the pandemic, the rates of hospitalization in the NICU were high because babies born to COVID-19 PCR (+) mothers were isolated until the PCR results were available. After hospitalization in the NICU, five (4.2%) of seven patients whose COVID-19 PCR sample was positive were lost.

One of the two babies whose first postnatal PCR test was positive remained asymptomatic and was discharged immediately. He died with the other due to progressive respiratory failure. No comorbidity was detected. PCR positivity was detected in five babies born to COVID-19 PCR (+) mothers on Postnatal Days 3–5. Four of the other five PCR positive babies died, and one was discharged.

The diagnosis was made when the control PCR tests were positive because the respiratory distress did not regress or new respiratory distress emerged in the patients. Three of the five cases were late preterm, one had transposition of the great arteries, and one had anal atresia. One of the cases was a term infant and had no known underlying disease. Respiratory distress in all cases increased gradually. Remdesivir, dexamethasone, and intravenous immunoglobulin treatments were given to a total of seven newborns. Five died, and two were discharged.

Demographic, clinical, and laboratory characteristics of newborns are summarized in Table 3.

## 4. Discussion

Pregnancy is a special condition that can have significant effects on a woman's body's biological systems. Cumulative, specific changes occur in pregnant women to facilitate immune system tolerance during pregnancy. These changes often put the mother's immune system in a downregulated state. As a result, pregnant women are often considered vulnerable to infectious diseases [9, 10].

COVID-19 has been linked to severe acute respiratory syndrome coronavirus 1 (SARS-CoV-1) which has shown a high incidence of adverse maternal and neonatal outcomes, including preterm delivery and intrauterine growth restriction (IUGR) in previous studies [11, 12]. A recent systematic review of pregnancies recently infected with coronavirus-related diseases revealed a high risk of miscarriage, pre-eclampsia, preterm birth, and perinatal death [13].

Allotey et al. [14] reported in their study as follows: Pre-existing comorbidities, nonwhite ethnicity, chronic hypertension, pre-existing diabetes, high maternal age, and high body mass index are risk factors for severe COVID-19 in pregnancy. In our study, however, there was no relationship between severe and treatment-requiring COVID-19 disease in diabetic or pre-eclamptic pregnancies, while the mortality rate was found to be higher in pre-eclamptic pregnancies. While almost half of all women were asymptomatic, fever, cough, fatigue, and muscle pain were the most commonly reported symptoms.

Lassi et al. in their systemic review showed that pregnant women with severe COVID-19 were approximately 3.7 years older, and the risk of severe COVID-19 was higher among women in a higher age group (> 35 years) [15]. In our study, the rate of receiving treatment due to COVID-19 was higher in pregnant women over the age of 35. However, in this age group, mortality during pregnancy was similar to the younger group.

In an observational study of 1219 pregnant patients who tested positive for SARS-CoV-2, increased rates of cesarean delivery, hypertensive pregnancy disorders, and preterm birth were seen in those with severe disease compared to asymptomatic patients [16].

Studies have revealed the relationship of COVID-19 with abortion [17, 18]. It was concluded that an important factor related to abortion in mothers with COVID-19 is inflammation and placental insufficiency due to the direct effect of the virus on the placenta [19, 20]. Therefore, fetal death may be a result of COVID-19 during pregnancy [21]. While first trimester abortion rates are observed at 10%–15% in studies, infant loss is 1%–2% in the second trimester [22]. In our study, second and third trimester baby losses were found to be 4%. The low socioeconomic level in the region where our hospital is located, the inadequacy of antenatal care, the high risk of pregnancies, and the low rate of vaccination during pregnancy may explain this situation.

A total of 11 (3.7%) pregnant deaths occurred. According to Lassi et al. [15] in their review, the pregnancy mortality rate was 2%. According to Mirbyk, Saghazedeh, and Rezaei [9] in his review, it was 0.5%. In our region, the rate of vaccination in the general population was low, and the rate of vaccination in pregnant women was much lower. According to the latest data, the rate of two-dose vaccination in our region is 64.3%.

In our study, almost 43.7% of all deliveries were performed by cesarean section; 23.2% had NST abnormalities. In addition, coronavirus transmission anxiety also played an important role in the delivery method.

Several observational studies have been published on the possibility of vertical transmission of SARS-CoV-2 during pregnancy [18]. However, there is no evidence that COVID-19 is transmitted to the fetus via the placenta in pregnancies [23]. Most SARS-CoV-2 infections identified among infants after birth are due to exposure to infected caregivers. However, it was observed that the risk of late postnatal transmission increased when babies were not separated from their infected mothers after birth [8].

Some studies found neonatal mortality rates (1.6%). Neonatal SARS-CoV-2 positivity was small (3.5%) and consistent in synthesis of other evidence [24, 25]. In a systematic review of developed countries, there are only three early neonatal deaths per 1000 live births [26]. Although PCR was (−) at the time of hospitalization in the first 3–5 days, four newborns who were later found (+) died during the follow-up. Neonatal mortality was found to be 1.7%.

Panda, Mishra, and Pathak reported fetal distress in mothers of SARS-CoV-2 in only three studies, and fetal distress was observed in 50 (18.2%) of 275 deliveries [27]. In 11 studies, 19 (1.14%) out of a total of 1666 baby births were stillbirths. A total of 103 newborns reported in 10 studies had perinatal asphyxia, and the pooled prevalence was 4.21% (2.36–6.50). In our study, perinatal asphyxia was found in 19 (6.3%) newborns. According to the data published by the Hypoxic Ischemic Encephalopathy Study Group of the Turkish Neonatology Society in Turkey in 2008, 93 infants in 19,857 live births were examined under the diagnosis of hypoxic-ischemic encephalopathy; the frequency was found to be 2.6 per thousand and 1.2% among patients hospitalized in intensive care units [28]. Recent studies have shown the accumulation of perivillous fibrin and multiple villous infarcts in the placenta of mothers infected with SARS-CoV-2 [29]. During the COVID-19 period, both the increase in intrauterine infant deaths and the increase in the rate of perinatal asphyxia can be explained.

Lassi et al. reported that almost half of all births were by cesarean section; a quarter of all births were preterm, less than a fifth had LBW, and a quarter reported admission to NICU in their meta-analysis [15]. While the rate of cesarean delivery was around 21% in our center in the pre-COVID-19 period, this rate increased to 50% in pregnant women with COVID-19. It is thought that the reason for this may be the clinician's concern about preventing virus transmission to the newborn.

In a study conducted in the field of neonatology, it was determined that the rates of cesarean section, prematurity, and low birth weight infants among COVID-19-positive pregnant individuals were 71.2%, 26.4%, and 12.8%, respectively [30].

In our study, 10% SGA, 22% low birth weight (< 2500 g), 26.1% prematurity (< 37 weeks), and 10.4% prematurity (≤ 32 weeks) were found. SARS-CoV-2-infected mothers (28.3%) had more preterm babies versus noninfected mothers (14.6%) as per the study by Gupta, Kumar, and Sharma [31], and Anand et al. reported the highest percentage of premature births and also the highest percentage of symptomatic mothers in this meta-analysis [32]. In a systematic review, preterm birth was the adverse outcome in SARS-CoV-2-infected pregnant mothers [13].

In the systemic review and meta-analysis by Van Baar et al., 120 studies involving pregnant women were evaluated [33]. No relationship has been found between SARS-COV-2 infection and intrauterine infant loss. Additionally, second and third trimester losses were not associated with COVID-19. In our study, losses in all three trimesters were similar. We interpreted this as the fact that some first trimester losses occurred outside our hospital and low socioeconomic levels prevented the recording of first trimester losses.

Previous studies reported that cesarean section rates have increased in pregnant women with COVID-19 [14, 34]. Schwartz et al. suggest that the increase in cesarean births may be due to SARS-CoV-2 placentitis causing fetal distress [35]. Göklü et al. reported that the presence of SARS-CoV-2 placentitis correlates with the development of placental dysfunction of acute/subacute onset. Also, it was considered that maternal clinical features are the main determinant of cesarean section rates in symptomatic pregnant women [36]. Similar to the literature, we found that the cesarean section rate (43.7%) was high in those who gave birth in our hospital.

Our studies have several limitations. First, although some of the pregnant women detected COVID-19 PCR (+) at various stages of pregnancy, information about the continuation of the pregnancy could not be obtained. Secondly, the vaccination rates in pregnant women were not clearly known locally, but it was observed that they were much lower than the vaccination rates in the general population. Third, it was observed that maternal and infant mortality peaked in the period when the Delta variant was dominant, but due to the retrospective nature of the study, the data could not be fully reached. Finally, amniotic fluids of newborns with postnatal COVID-19 PCR (+) could not be evaluated.

## 5. Conclusions

Our current study suggests that the clinical features and prognosis of pregnant women with COVID-19 are worse than those of the general population. Especially in our region, where there is no vaccination during pregnancy and antenatal follow-up is not good, the loss of pregnant and intrauterine infant deaths has increased dramatically. In the general newborn population, there has been an increase in the rate of neonatal hospitalization due to prematurity, low birth weight, and perinatal asphyxia due to intrauterine placental insufficiency rather than problems directly related to viral load. In addition, comorbidities have been observed in deaths associated with COVID-19 in newborns.

## Figures and Tables

**Table 1:** Clinical and laboratory characteristics of pregnant women.

**COVID-19 PCR (+) pregnant women**	**n** = 327
Medical treatment for COVID-19, *n* (%)	93 (28.4)
Presence of at least one COVID-19 symptom, *n* (%)	185 (56.6%)
Gestational diabetes, *n* (%)	10 (3)
Preeclampsia, *n* (%)	25 (7.6)
Eclampsia, *n* (%)	4 (1.2)
Clinical chorioamnionitis, *n* (%)	11 (3.4)
Pregnant over 35 years, *n* (%)	53 (16.2)
Maternal death, *n* (%)	11 (3.4)
Need for intensive care due to COVID-19, *n* (%)	54 (16.5)

**Table 2:** Comparison of clinical and demographic characteristics of the groups.

	**Group 1** ^∗^ **N** = 11	**Group 2** ^∗∗^ **N** = 316	**p** **value**
Age, median (IQR 25–75)	33 (27–35)	28 (25–33)	0.087
Gestational diabetes, *n* (%)	1 (9)	9 (2.8)	0.28
Preeclampsia, *n* (%)	3 (27.3)	22 (7)	0.034
Eclampsia, *n* (%)	0	3 (0.9)	0.908
Intrauterine fetal demise, *n* (%)	2 (18.2)	19 (60)	0.15
Abnormal NST^∗∗∗^, *n* (%)	6 (54.5)	70 (22.2)	0.01
Perinatal asphyxia, *n* (%)	4 (36)	15 (4.7)	0.027
Birth weight, median (IQR 25–75)	2250 (1825–2450)	3000 (2500–3370)	0.01

^*^Group 1: pregnant women who lost their lives during the COVID-19 working process.

^**^Group 2: pregnant women who survived COVID-19 in good health.

^***^Nonstress test.

**Table 3:** Clinical and laboratory characteristics of neonates.

C/S, *n* (%)∗	131 (42.8)
Male, *n* (%)∗	154 (50.3)
Prematurity (< 37 gw), *n* (%)∗	80 (26.1)
Prematurity (≤ 32 gw), *n* (%)∗	32 (10.4)
Low birth weight (< 2500 g), *n* (%)∗	66 (22)
Small for gestational age, *n*(%)∗	23 (7.5)
Apgar at 1 min, median (min–max)∗	8 (1–9)
Apgar at 5 min, median (min–max)∗	9 (2–10)
Perinatal asphyxia, *n* (%)∗	19 (6.2)
Total neonatal death, *n* (%)∗	13 (4.2)
COVID-19-related neonatal mortality, *n* (%)∗	5 (1.6)
COVID-19 PCR (+) at birth∗	2 (0.7)
Intrauterine fetal demise, *n* (%)∗∗	21 (6.4)
Abortus, *n* (%)∗∗	8 (2.4)
Stillbirth, *n* (%)∗∗	13 (4)
NICU admission, *n* (%)∗	119 (38.9)
Isolation, *n* (%)∗	49 (16)

∗Number of live births: 306.

∗∗Total number of pregnancies: 327.

## Data Availability

I confirm that I have included a Data Availability Statement in my manuscript.
